# Low Serum Levels of Zonulin in Patients with HCV-Infected Chronic Liver Diseases

**DOI:** 10.5005/jp-journals-10018-1275

**Published:** 2019-02-01

**Authors:** Tomohiro Akao, Ayumi Morita, Morikazu Onji, Teruki Miyake, Ryouzi Watanabe, Takahide Uehara, Keitarou Kawasaki, Jiro Miyaike, Masaki Oomoto

**Affiliations:** 1 Saiseikai Imabari Hospital, Kitamura, Imabari, Ehime, Japan; 2 Emeritus Professor of Ehime University, Ehime University Graduate School of Medicine, Shitsukawa, Toon, Ehime, Japan; 3 Department of Gastroenterology and Metabology, Ehime University Graduate School of Medicine, Shitsukawa, Toon, Ehime, Japan

**Keywords:** Intestinal permeability, Leaky gut syndrome, Liver cirrhosis, Zonulin

## Abstract

**Aim:**

The aim of the study was to assess the implication of Zonulin, a mediator protein synthesized by intestine and the liver,in patients with chronic liver diseases.

**Materials and methods:**

Twenty-six patients with chronic liver diseases due to hepatitis C virus (HCV) and hepatitis B virus (HBV) were enrolled in this study. Out of total 26 patients, 17 were diagnosed as chronic hepatitis (CH) and 9 were patients with liver cirrhosis (LC). Twenty-four of these patients were infected with hepatitis C virus (HCV) and the rest two by hepatitis B virus (HBV). The study was conducted at Saiseikai-Imabari Hospital, Imabari, Ehime, Japan. Serum levels of Zonulin along with different parameters of liver function test were measured in all patients and comparative analyses were accomplished.

**Results:**

The serum levels of Zonulin were significantly lower in CH patients compared to controls (p<0.001). Also, the levels of Zonulin were significantly lower in patients with LC compared to CH and normal controls (p<0.001). Further analysis revealed that serum Zonulin was significantly lower in patients with LC having ascites than those without ascites (p <0.05). There was a significant correlation of serum levels of Zonulin with platelet count, cholinesterase, and albumin in patients with chronic liver diseases.

**Discussion:**

Decreased levels of Zonulin may be related to impaired production of this mediator in the diseased liver. It will be tempting to assess the regulation of Zonulin in the liver, a production site of the mediator.

**Abbreviations:**

LC: Liver cirrhosis, CH: Chronic hepatitis, HCV: Hepatitis C virus, HBV: Hepatitis B virus, LGS: Leaky gut syndrome

**How to cite this article:** Akao T, Morita A, Onji M, Miyake T, Watanabe R, Uehara T, Kawasaki K, Miyaike J, Oomoto M. Low Serum Levels of Zonulin in Patients with HCV-Infected Chronic Liver Diseases. Euroasian J Hepatogastroenterol, 2018;8(2):112-115.

## INTRODUCTION

Increased intestinal permeability is associated with pathogenesis of various diseases, especially in cholera-induced watery diarrhea.^1^ Dr. Fasano and his colleagues found that cells in the human intestine produce a protein that is almost identical to the zonula occludens toxin, a toxin related to pathogenesis of cholera, and they named it Zonulin.^2-4^ Zonulin is a protein, synthesized in intestinal and liver cells, that reversibly regulates intestinal permeability. Dr. Fasano’s group then isolated Zonulin from human intestines and found it to increase intestinal permeability in primates.^2-4^

The role of Zonulinin maintenance of tight junction has been well explored and established.^1-4^ In addition, Zonulin has been found be associated with various pathological conditions such as autoimmunity and malignancies, possibly by their capacity to modulate tight junction and regulating releasing of various antigens and mediators from gut to circulation.^5-7^ However, other prospective function of Zonulin is yet to be explored. Specifically, Zonulin have been found to be associated with the pathogenesis of coeliac disease (CD) and type 1 diabetes, two autoimmune conditions in which the finely tuned regulation of intestinal tight junction permeability is lostalong with other pathogenetic processes.^5-7^

Although Zonulin is produced by liver, little is known about the role of Zonulin in pathological conditions of liver. Liver is one of the vital organs that has dominant role during synthesis, metabolism, and excretion in physiological conditions. Also, it undergoes inflammation, fibrosis, and carcinogenesis due to effects of viruses and other factors. These facts indicate that there may be a relation between Zonulin and different types of liver diseases.

The study presented here have been undertaken to assess the association and role of Zonulin in liver diseases, if any.

## MATERIALS AND METHODS

### Study Subjects

Twenty-six patients with chronic liver diseases attending SaiseikaiImabari Hospital, Imabari, Ehime, Japan were enrolled in this study. Twenty-four of them infected with HCV and rest 2 by HBV. The diagnosis of chronic liver diseases was made based on the recommendations of international professional liver organizations (American Association for the Study of the Liver, AASLD; European Association for the Study of the Liver, EASL and Asia-Pacific Association for the Study of the Liver, APASL) and Japanese Society of Hepatology. The patients with chronic hepatitis (CH) had biochemical and virological evidences of CH and the extent of FIB-4 index was between 1.45 to 3.25. The patients with liver cirrhosis (LC) had nodular liver as confirmed by imaging and FIB-4 index was more than 3.25. In addition, 27 healthy subjects attending the hospital for medical checkup were included as controls. The subjects of control group had normal alanine ami-notransferase (ALT) and aspartate transaminase (AST) and FIB-4 index of less than 1.45 with no previous history of liver diseases. This study was approved by the Ethics Committee of SaiseikaiImabari Hospital July 26, 2018 (approval number I30-7) and all patients and controls provided their consent to the study.

### Blood Samples and Measurement

Blood samples were collected from all patients. Parameters other than Zonulin were immediately measured after centrifugation and the remaining serum was stored frozen at -70. Measurement of serum Zonulin was carried out within 3 months from preservation. For measurement of Zonulin in serum, Zonulin ELISA Kit manufactured by ImmunDiagnostik(Shirley, NY) was used. Using the control substance in the kit, accuracy control was performed using the management data attached to the kit.

Along with the serum Zonulin measurement, the levels of AST, ALT cholinesterase (CHE), albumin (ALB) and platelets (PLT) were assessed. PT-INR were calculated as well. We used LABSPECT008 (Hitachi High-Tech Fielding Company, Tokyo, Japan) for AST, ALT, CHE and ALB and XN-9000 (Sysmex Corporation, Hyogo, Japan) for PLT and CS-5100 (Sysmex Corporation, Hyogo, Japan) for PT-INR and ARCHITECTi2000SR (Abbott Japan Corporation, Tokyo, Japan) for HBsAg, HCVAb and cobas Ampliprep and cobas TaqMan (Roche Diagnostics corporation, Tokyo, Japan) for HCV-RNA.

### Statistical Analysis

In the statistical analysis methods, the reference range was calculated using MCP-TQA by Sysmex Corporation, and the significant difference test was by Student’s T test.

## RESULTS

### Accuracy Control

Because of double measurement of the management serum Low and High in the kit, the SDI was -0.62 to 1.19, within the control standard range within ± 2 SD. Because of calculating reference standard range of Zonulin of the control group, reference standard range of serum levels of Zonulin was 25.2 to 66.5 ng/mL, the distribution was 1/ SQRT (X), there was no gender difference (p = 0.124).

### Comparison of Zonulin Concentrations in each Group

**Fig. 1:**
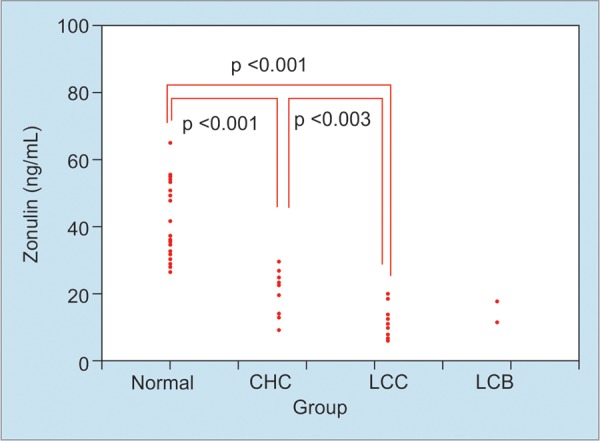
Comparison of Zonulin in each group

The serum levels of Zonulin in Controls were 40.26 ± 10.23 ng/ml. This was significantly decreased in CHC (20.10 ± 7.09 ng/mL), and LC (11.99 ± 4.92 ng/mL). Also, the levels of Zonulin were significantly lower in LC than CHC (p < 0.001, p = 0.003) (Fig. 1). Two patients were LC due to HBV. These two patients also had decreased Zonulin (Fig. 1). It may be a fact that decreased Zonulin is a property of LC, not their etiology.

**Table 1:** **Table 1:** Comparison of zonulin with or without ascites

		*Without ascites*				*With ascites*			
*Sl. No.*		*FIB4*		*Zonulin*		*FIB4*		*Zonulin*	
1		4.46		20.3		13.45		5.7	
2		3.33		18.6		6.03		10.4	
3		3.62		11.0		23.63		8.4	
4		4.15		10.2		7.95		7.3	
5		10.57		9.6		706.60		7.9	
6		3.58		18.4		7.39		6.6	
7		4.11		12.9		18.37		18.9	
8		5.38		13.9					
Average		4.900		14.34		111.918		9.31	
Standard deviation		2.378		4.21		262.309		4.47	

Verification: FIB4 p = 0.004, zonulin p = 0.043

### Comparison of serum levels of Zonulin in groups with and without ascites in LC

Among LC patients, the levels of Zonulin was significantly lower in patients with ascites (9.31±4.47 ng/mL) compared to those in patients without ascites (14.34 ± 4.21 ng/mL (p = 0.042) (Table 1).

### Correlation with PLT, CHE, ALB and PT-INR

**Figs 2A to D:**
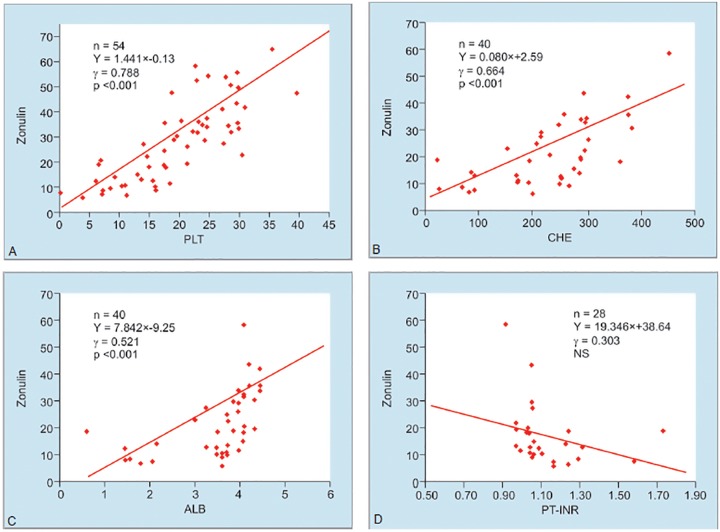
Correlation between the serum levels of zonulin and PLT, CHE, ALB, PT-INR

The levels of Zonulin showed correlation with PLT (y = 1.441 x - 0.13, = 0.788, p <0.001), CHE (y = 0.080 x + 2.59, = 0.664, p <0.001) and ALB (y = 7.842 x - 9.25, = 0.521, p <0.001) (Fig. 2).

## DISCUSSION

In the intestinal mucosa, Zonulin inhibits the entry of food antigens, bacteria, pathogens and toxins while selective absorption of necessary nutrients proceeds. In intestinal mucosal leak syndrome (LGS), chemical substances and harmful substances leak into the body due to the increases of the intestinal permeability. For these reasons, the coordination between mucosal cells is important, and Zonulin should be the important protein that is responsible for it. Zonulin is a precursor of hapto-globulin produced in the liver and gastrointestinal tract, a protein that widens the gap between intestinal cells.^1-4^ The concept of Zonulin activity in tight junction has been provisionally supported as Zonulin in increased celiac disease, type I diabetes, multiple sclerosis, rhinitis spon-dylitis, asthma, ulcerative colitis.^5-7^ Recently, increased levels of Zonulin have been reported in patients with fatty liver.^8^

From these pretexts, the data provided in this article seems to be bit surprising. We have found that Zonulim is significantly decreased in CH patients compared to normal controls and also in LC patients compared to both CH patients and controls. Although these data have not supported a notion that increased Zonulin is a factor related to different pathologies, it is not new to show either no involvement or different forms of involvement of Zonulin in different pathological conditions. Wex et al have shown that zonulin is not increased in the cardiac and esophageal mucosa of patients with gastroesophageal reflux disease.^9^ In line with this, Gereds et al. proposed that Zonulin may not be a marker of autoimmunity in patients with psoriasis.^10^ Lukaszyk et al. also concluded that Zonulin cannot be considered as an inflammatory marker in chronic kidney disease (CKD).^11^ It does not play a role in the disturbances of iron metabolism in CKD.

In our study, we also found decreased levels of Zonulin along the progression of liver diseases. Although the exact mechanisms underlying this remains to be explored, we have proposed a new arena of Zonulin study in which its functional activity as well its production by liver should be clearly dissected. Ohlsson et al. proposed about Zonulin level with disease risk and pathogenesis.^12^ He mentioned that higher levels of serum Zonulin may rather be associated with increased risk of obesity and hyperlipidemia, than with gastrointestinal symptoms or disease manifestations. Thus, there may be dichotomy of level of Zonulin and functional implications. This is specially manifested when as we found that the levels of Zonulin were decreased in patients with decompensated cirrhosis. In all patients without ascites, the levels were more than 9.6 ng/mL., whereas, only 2 of 7 patients with ascites had Zonulin levels more 9.6. These facts required to be studied in more details as there may be therapeutic implication of Zonulin in decompensated cirrhosis. The most important factor would be if the decreased levels of Zonulin represents an effect of hepatic injury in decom-pensated cirrhosis.
